# Epirubicin–vinorelbine *vs* FEC100 for node-positive, early breast cancer: French Adjuvant Study Group 09 trial

**DOI:** 10.1038/sj.bjc.6603773

**Published:** 2007-05-15

**Authors:** P Kerbrat, H Roché, J Bonneterre, C Veyret, A Lortholary, A Monnier, P Fumoleau, P Fargeot, M Namer, P Chollet, M-J Goudier, B Audhuy, H Simon, P Montcuquet, J-C Eymard, S Walter, P Clavère, J-P Guastalla

**Affiliations:** 1 Department of Medical Oncology, Centre Eugène Marquis, Université de Rennes, Rue de la Bataille Flandres-Dunkerque, CS 44229, 35042 Rennes, France; 2 Department of Medical Oncology, Institut Claudius Régaud, 20-24 rue du Pont Saint-Pierre, 31052 Toulouse, France; 3 Department of Medical Oncology, Centre Oscar Lambret, 3 rue Frédéric Combemale, 59020 Lille, France; 4 Department of Medical Oncology, Centre Henri Becquerel, Rue d’Amiens, 76038 Rouen, France; 5 Department of Medical Oncology, Centre Paul Papin, rue Moll, 49000 Angers, France; 6 Department of Radiotherapy and Medical Oncology, Centre Hospitalier André Boulloche, 25209 Montbéliard, France; 7 Department of Medical Oncology, Centre René Gauducheau, Boulevard Jacques Monod, 44805 Nantes St-Herblain, France; 8 Department of Medical Oncology, Centre Georges-François Leclerc, 1 rue du Professeur Marion, BP 77980, 21079 Dijon, France; 9 Department of Medical Oncology, Centre Antoine Lacassagne, 33 avenue de Valombrose, 06189 Nice, France; 10 Department of Medical Oncology, Centre Jean Perrin, 58 rue Montalembert, 63011 Clermont-Ferrand, France; 11 Department of Medical Oncology, Centre Hospitalier de Bretagne Sud, BP 2233, 56322 Lorient, France; 12 Department of Medical Oncology, Centre Hospitalier Louis Pasteur, 39 avenue de la Liberté, 68021 Colmar, France; 13 Department of Medical Oncology, Centre Hospitalier Universitaire Augustin Morvan, 5 avenue Foch, 29285 Brest, France; 14 Department of Medical Oncology, CliniqueSaint-Vincent, 40 chemin de Tilleroyes, 25000 Besançon, France; 15 Department of Medical Oncology, Institut Jean Godinot, 1 avenue du Général Koenig, 51056 Reims, France; 16 Department of Medical Oncology, Hôpital Notre-Dame de Bon Secours, 1 place Philippe de Vigneulles, 57038 Metz, France; 17 Department of Medical Oncology, Centre Hospitalier Universitaire Dupuytren, 2 avenue Martin Luther-King, 87042 Limoges, France; 18 Department of Medical Oncology, Centre Léon Bérard, 28 rue Laënnec, 69008 Lyon, France

**Keywords:** early breast cancer, adjuvant chemotherapy, epirubicin, vinorelbine

## Abstract

The aim of the study was to compare our reference adjuvant chemotherapy, FEC100 (fluorouracil 500 mg m^−2^, epirubicin 100 mg m^−2^ and cyclophosphamide 500 mg m^−2^, six cycles every 21 days), to an epirubicin–vinorelbine (Epi-Vnr) combination for early, poor-prognosis breast cancer patients. Patients (482) were randomised to receive FEC100, or Epi-Vnr (epirubicin 50 mg m^−2^ day 1 and vinorelbine 25 mg m^−2^, days 1 and 8, six cycles every 21 days). The 7-year disease-free survival rates were 59.4 and 58.8%, respectively (*P*=0.47). The relative dose intensity of planned epirubicin doses was 89.1% with FEC100 and 88.9% with Epi-Vnr. There were significantly more grades 3–4 neutropenia (*P*=0.009) with Epi-Vnr, and significantly more nausea-vomiting (*P*<0.0001), stomatitis (*P*=0.0007) and alopecia (*P*<0.0001) with FEC100. No cases of congestive heart failure were reported, whereas four decreases in left ventricular ejection fraction occurred after FEC100 and five after Epi-Vnr. One case of acute myeloblastic leukaemia was registered in the FEC100 arm. After 7 years of follow-up, there was no difference between treatment arms. Epi-Vnr regimen provided a good efficacy in such poor-prognosis breast cancer patients, and could be an alternative to FEC100, taking into account respective safety profiles of both regimens.

Successive overviews by the Early Breast Cancer Trialists’ Collaborative Group (EBCTCG) have well established the benefit of adjuvant chemotherapy in node-positive breast cancer patients irrespective of age as well as the pivotal role of anthracycline-based chemotherapy, which significantly reduced annual rates of relapse and death compared with the combination of cyclophosphamide, methotrexate and fluorouracil (Early Breast Cancer Trialists’ Collaborative Group (EBCTCG), 2005). After 10 years of follow-up, the French Adjuvant Study Group (FASG)-05 trial has showed previously that six cycles of FEC100 (fluorouracil 500 mg m^−2^, epirubicin 100 mg m^−2^ and cyclophosphamide 500 mg^−2^) significantly improved disease-free survival (DFS) and overall survival (OS) compared with FEC50 (same regimen with epirubicin 50 mg m^−2^), in poor-prognosis, node-positive breast cancer patients (Bonneterre *et al*, 2005). On the basis of results of FASG-05 trial, the FEC100 regimen was considered one of the reference treatments for node-positive breast cancer.

In first-line metastatic breast cancer treatment, single-agent therapy with vinorelbine has shown overall response rates varying from 35 to 60% with a good clinical tolerance (Canobbio *et al*, 1989; Fumoleau *et al*, 1993; García-Conde *et al*, 1994; Weber *et al*, 1995). Thereby, it was logical to combine vinorelbine and anthracycline to evaluate their activity in advanced breast cancer. Impressive results have been obtained through an every 3-week schedule of vinorelbine 25 mg m^−2^, days 1 and 8, plus doxorubicin 50 mg m^−2^ on day 1, producing an objective response rate of 74% (complete response 21%) and a median survival time of 27.5 months (Spielmann *et al*, 1994), but at the price of a high level of cardiac toxicity as 10% of patients experienced grades 2–4 treatment-related cardiotoxicity. In a phase III trial, comparing vinorelbine–doxorubicin and FAC (fluorouracil 500 mg m^−2^, doxorubicin 50 mg m^−2^ and cyclophosphamide 50 mg^−2^), the efficacy of both regimens was similar, whereas vinorelbine–doxorubicin regimen was more active in the subset of patients with visceral metastatic disease, especially liver involvement (Blajman *et al*, 1999). Giving the better safety profile of epirubicin compared with doxorubicin in terms of haematologic and cardiac toxicities (Torti *et al*, 1986; Mouridsen, 1990), and the similar efficacy of both anthracyclines when used at equimolar doses of 50 mg m^−2^ (French Epirubicin Study Group, 1988; Italian Multicentre Breast Study with Epirubicin, 1988), the replacement of doxorubicin by epirubicin could be of interest. Subsequently, the FASG-05 trial demonstrated a significant superiority of FEC100 over FEC50 regimen in adjuvant setting (Bonneterre *et al*, 2005). The use of FEC100 regimen led to 1% of congestive heart failure (CHF) after 10 years of follow-up, without cardiac death. Thereby, we considered six cycles of FEC100 as our standard adjuvant chemotherapy for node-positive breast cancer patients.

In 1993, we initiated a randomised phase III trial, FASG-09, to compare our reference adjuvant chemotherapy regimen, FEC100, to an Epi-Vnr combination in poor-prognosis, node-positive early breast cancer patients. The primary end point was the DFS, and secondary end points were OS and safety.

## PATIENTS AND METHODS

### Study population

Women eligible for the study were between 18 and 64 years of age, and had undergone primary surgery (modified mastectomy or tumorectomy) plus axillary dissection for unilateral, operable carcinoma of the breast. Patients had to present with histologically proven axillary lymph node involvement (at least five nodes removed), and either more than three positive nodes or one to three positive nodes plus SBR grade ⩾2 and HR-negative tumour (estrogen (ER) and progesterone (PgR) receptors). Main eligibility criteria included the World Health Organisation (WHO) performance status ⩽2, adequate haematologic (granulocyte count ⩾2 × 10^9^ l^−1^ and platelets count ⩾100 × 10^9^ l^−1^), hepatic (bilirubin⩽35 *μ*mol l^−1^) and renal (serum creatinine ⩽130 *μ*mol l^−1^) tests, and no cardiac dysfunction (left ventricular ejection fraction (LVEF) ⩾50%). Patients were excluded from the study if they had evidence of metastases, documented history of cardiac disease contraindicating anthracyclines, previous cancer (except treated basal cell and squamous cell carcinoma of the skin or cancer of the uterine cervix), previous radiation therapy, hormonotherapy or chemotherapy for breast cancer or were greater than 42 days from initial breast cancer surgery.

Potentially eligible patients underwent bone scan, chest X-ray, abdominal ultrasound or CT scan, and contralateral mammography. Patients had a cardiac assessment consisting of an electrocardiogram (ECG) and a LVEF measurement at rest by radioisotopic or echographic methods. Written informed consent was obtained before randomisation. The protocol was reviewed and approved by the Ethics Committee/Institutional Review Board, and the study was conducted according to the Declaration of Helsinki and French Health Authorities requirements.

### Study design

This was a randomised, multicenter and open-label phase III study. Randomisation procedures were centralized and balanced per block. Patients were assigned to receive FEC100 (fluorouracil 500 mg m^−2^, epirubicin 100 mg m^−2^ and cyclophosphamide 500 mg m^−2^ intravenously on day 1, every 21 days for six cycles) or Epi-Vnr (epirubicin 50 mg m^−2^ intravenously on day 1 and vinorelbine 25 mg m^−2^ intravenously on days 1 and 8, every 21 days for six cycles). Stratification was by the number of positive auxillary nodes (1–3, 4–9 and ⩾10) and centre. Primary prophylaxis with granulocyte colony-stimulating factors (G-CSF) and antibiotics was prohibited. Antiemetics were prescribed routinely before each cycle. The allocated treatment was started within 42 days after initial surgery. An absolute granulocyte count <2 × 10^9^ l^−1^ and/or a platelet count <100 × 10^9^ l^−1^ on day 21 led to a treatment delay of at least 1 week. Treatment was stopped if haematologic recovery took more than 3 weeks beyond day 21. The epirubicin dose was reduced by 50%, if serum bilirubin levels were 35–50 *μ*mol l^−1^ and treatment was stopped if bilirubin levels exceeded 50 *μ*mol l^−1^.

Tamoxifen (30 mg day^−1^) was started at the first chemotherapy cycle and continued for 3 years in postmenopausal women. For HR-negative patients, treatment with tamoxifen was given at the investigator's discretion, but the policy had to be similar for both arms at each centre. Radiotherapy was initiated within 4 weeks after the last cycle of chemotherapy and consisted of radiation to the chest wall, supraclavicular area, internal mammary chain and auxillary area (in case of pN1 tumour). In patients who had undergone breast-conserving surgery, a complementary boost was delivered to the breast. Radiotherapy procedures had to be similar for both arms at a given centre.

The tolerability of chemotherapy was evaluated before each cycle, an ECG and an absolute blood count were performed on day 21, and non-haematologic toxicity was evaluated during the period between cycles. Toxicity was graded according to the WHO criteria. It was recommended to assess LVEF within 3–4 weeks after the last chemotherapy cycle. A decrease in LVEF was defined as an absolute value below 50%, and/or a relative decline of more than 20% compared with baseline value. Additional assessment of LVEF was left at the discretion of each investigator. Patients underwent clinical and biochemical assessments every 6 months during a 5-year follow-up period, and yearly thereafter. Imaging studies (mammography, chest X-ray, liver ultrasound and bone scan) were performed yearly during a 5-year follow-up period and every 2 years thereafter. Patients were followed until death.

### Statistical analysis

The primary end point was the 5-year DFS defined as the time from randomisation until the first relapse (local, regional and/or distant). A contralateral breast cancer was considered a new primary malignancy. This trial was designed to detect a 10% difference in DFS with a power of 80%, and a two-sided type I error of 5%. These hypotheses required enroling 460 patients. Data were analysed according to the intention-to-treat (ITT) principle, using SPSS software (SPSS Inc., Chicago, IL, USA). Secondary end points were OS defined as the time from randomisation until death from any cause and safety. Patients who received at least one dose of study drug were analysed for safety.

The *χ*^2^ test was used to compare baseline categorical variables and incidence of adverse events between treatment arms. Continuous variables were compared by using analysis of variance. The relative dose intensity (RDI) was calculated based on the ratio of the drug dose actually delivered in the originally expected time to the expected dose in the expected time. The DFS and OS rates were calculated by the Kaplan–Meier method, and were compared using a log-rank test. A multivariate analysis (Cox regression model) was adjusted for age, menopausal status, surgery, SBR grade, histological tumour size, number of positive auxillary lymph nodes and HR status.

## RESULTS

### Patient characteristics

Between June 1993 and April 1998, 18 French centres enrolled 482 patients (241 in FEC100 and 241 in Epi-Vnr). Of these women, seven were lost to follow-up at the time of randomisation and were censored at this date, and four presented with an initial metastatic disease. Efficacy analysis involved 471 patients (235 in FEC100 and 236 in Epi-Vnr). The compliance and safety analyses were performed on 469 treated patients (236 in FEC100 and 233 in Epi-Vnr). Baseline characteristics were well balanced between treatment arms (Table 1). However, the exclusive comparison of ductal and lobular carcinomas exhibited a significant higher rate of lobular carcinomas in the FEC100 arm (*P*=0.03).

### Treatment

Among the 469 treated patients, six treatment cycles were completed by 94.9% of patients in the FEC100 group and by 96.1% of the patients in the Epi-Vnr group (*P*=0.36). Twenty-one patients stopped prematurely the treatment protocol (12 in FEC100 and 9 in Epi-Vnr). Among these treatment interruptions, two patients died in the FEC100 arm: one from a septic shock and one from a rhythm disorder related to a hypokaliemia. The remaining reasons were haematologic toxicity and/or infectious complications (*n*=5), digestive toxicity (*n*=6), pulmonary embolism (*n*=2), patient refusal (*n*=3), cardiac rhythm disorders (*n*=1), late discovery of initial liver metastases (*n*=1) and unknown reason (*n*=1). Treatment compliance and doses are summarised in Table 2. There were significantly more cycles delayed beyond 24 days in the Epi-Vnr arm (30.7 *vs* 25.6%, *P*=0.0028), and the day 8 infusion of vinorelbine was not delivered in 14 cycles (1%). The epirubicin RDI was not different between treatment arms (*P*=0.87), although the dose density was twice in the FEC100 arm.

Tamoxifen was prescribed in 87 patients (36.9%) of FEC100 group and in 84 patients (36.1%) of Epi-Vnr group. Among patients who received tamoxifen, 25 (10.6%) and 31 (13.3%) were HR-negative, respectively. Three premenopausal patients received tamoxifen (one in FEC100 and two in Epi-Vnr). Radiotherapy was delivered in 227 (96.2%) and 229 (98.3%) treated patients, respectively.

### DFS and OS

**Figure 1:**
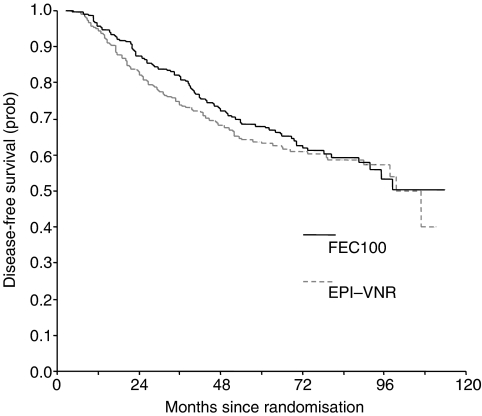
Disease-free survival curves.

The median follow-up time from randomisation was 78 months (range: 2–113). At the cut-off date for analysis, 89 patients (37.9%) had relapsed in the FEC100 group and 96 (40.7%) in the Epi-Vnr group (Table 3). The 7-year DFS rates were 59.4% (95% confidence interval (95% CI), 52.5–66.3%) with FEC100 and 58.8% (95% CI, 52.1–65.5%) (*P*=0.47; Table 4 and Figure 1) in Epi-Vnr. The incidence of local relapse was 8.1 and 10.6%, respectively. There was no difference in the pattern of recurrences, and the most common site of relapse was bone (39.3 and 42.7%, respectively). A plurimetastatic disease was reported in 28.1 and 33.3% of patients, respectively (*P*=0.44). Patients receiving tamoxifen showed improved DFS rates, irrespective of chemotherapy regimen (64.9 *vs* 55.7%, *P*=0.009). The Cox proportional hazards model showed that modified mastectomy and histological tumour size >20 mm were independent prognostic factors of relapse, knowing that both factors were significantly correlated (Table 4). In this model, the comparison between treatment arms remained not significant.

**Figure 2:**
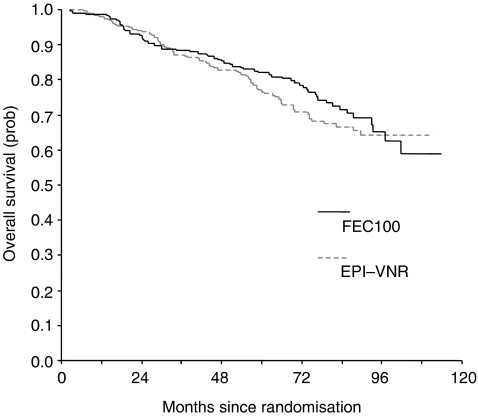
Overall survival curves.

There were 133 deaths involving 62 patients (26.4%) in the FEC100 arm and 71 patients (30.1%) in the Epi-Vnr arm (Table 3). The 7-year OS rates were 71.5% (95% CI, 64.8–78.2%) with FEC100 and 66.7% (95% CI, 60–73.4%) (*P*=0.38) with Epi-Vnr (Figure 2). All but 16 deaths (eight in FEC100 and eight in Epi-Vnr) were owing to progression of the disease.

### Acute and delayed toxicities

Toxicity was evaluated in 469 patients according to the WHO criteria. Adverse events experienced per patient are described in Table 5. The incidence of grades 3–4 neutropenia on day 21 was more frequent with Epi-Vnr (*P*=0.009). Although no significant difference was observed in terms of infection, one patient died from a septic shock in the FEC100 arm secondary to a poorly monitored febrile neutropenia at home. One case of grade 1 thrombocytopenia occurred with FEC100 and one case of grade 2 with Epi-Vnr. There were significantly more nausea-vomiting, stomatitis and alopecia with FEC100.

During chemotherapy, 20 cardiac abnormalities were diagnosed (10 in FEC100 and 10 in Epi-Vnr). Among these 20 cases, one consisted of a decrease in LVEF below 50% (Epi-Vnr arm). The other cases were rhythm disorders, of which one patient died secondary to a hypokaliemia.

After FEC100, in free of disease patients during the follow-up period, two patients developed a decrease in LVEF below 50%, one died from a mitral insufficiency and one had T-wave abnormalities diagnosed on ECG. After Epi-Vnr, four patients presented with a decrease in LVEF below 50% and one developed a cardiomyopathy. In patients who relapsed, two had a decrease in LVEF consecutively to a first-line chemotherapy with epirubicin (cumulative dose, 200 mg m^−2^) and mitoxantrone (cumulative dose, 39 mg m^−2^). Both patients had received FEC100 as adjuvant chemotherapy. Overall, no cases of CHF were reported.

### Second malignancies

Twenty patients developed a contralateral breast cancer (10 in FEC100 and 10 in Epi-Vnr; Table 3). Thirteen patients developed a second cancer (eight in FEC100 and five in Epi-Vnr; Table 3). One case of acute myeloblastic leukaemia FAB 4 with del(16q) has occurred 81 months after receiving FEC100, and 1 year after the initiation of a hormonotherapy for bone progression. This patient was still alive at the last contact date. Remaining cases were chronic lymphocytic leukaemia (*n*=1), endometrial carcinoma (*n*=1), uterine cervix carcinoma (*n*=2), colorectal cancer (*n*=5), sarcoma (*n*=1), head and neck carcinoma (*n*=1), and basal cell carcinoma of skin (*n*=1).

## DISCUSSION

Our results demonstrated an absence of difference between FEC100 and Epi-Vnr regimens for both DFS and OS in poor-prognosis and node-positive breast cancer. These results are slightly different from our initial report presented after 5 years of follow-up, in which a trend in a better DFS with FEC100 regimen was reported, providing 5-year DFS rates of 70.9 *vs* 63.8% with Epi-Vnr (*P*=0.07; Kerbrat *et al*, 2002). This observation of a decrease in the efficacy of FEC100 during the course of time has been also highlighted in the FASG-05 trial, as absolute differences between FEC50 and FEC100 regimens were 11.5% after 5 years of follow-up and 5.4% after 10 years (French Adjuvant Study Group, 2001; Bonneterre *et al*, 2005). This could mean that the efficacy of FEC100 regimen is greater within first years following the onset of adjuvant chemotherapy. Moreover, the design of the present trial, with a power of 80%, was by definition insufficient to detect a difference between treatment arms.

One could argue that our schedule of tamoxifen 30 mg day^−1^ for 3 years given in postmenopausal women does not square to the current standard of care for tamoxifen. We have to consider that this trial was initiated at the beginning of 1993. At this period, the benefit of tamoxifen in premenopausal, hormone-receptor-positive patients was not established. This has been clearly demonstrated in the EBCTCG overview presented in 1995 and published in 1998 (Early Breast Cancer Trialists’ Collaborative Group (EBCTCG), 1998). Since 1995, the duration of tamoxifen treatment was extended to 5 years because of the EBCTCG overview results. This overview concluded that 5 years was better than 1 or 2 years (Early Breast Cancer Trialists’ Collaborative Group (EBCTCG), 1998). To date, no data allows to conclude to the inferiority of 3 or 4 years, and to provide a difference between 20 and 30 mg day^−1^ of tamoxifen. Nevertheless, patients treated in the FASG-09 trial received similar tamoxifen therapy, which did not modify our conclusions.

The compliance to chemotherapy was similar between both arms. Noteworthy, the RDI of epirubicin was 89.1% with FEC100 and 88.9% with Epi-Vnr, in which epirubicin was administered at 50 mg m^−2^. The epirubicin RDI observed with FEC100 was close to the 91% RDI reported in the FASG-05 trial (Bonneterre *et al*, 2005). On the contrary, the RDI of epirubicin 50 mg m^−2^ in the Epi-Vnr regimen was lower than those of 94% previously reported using six cycles of FEC50 in the FASG-05 trial (Bonneterre *et al*, 2005). The additive haematologic toxicity of epirubicin combined with vinorelbine was probably the main explanation of this decrease in RDI. Indeed, Epi-Vnr regimen led to a significant increase in grades 3–4 neutropenia on day 21 compared with FEC100 (24.0 *vs* 17.4%; *P*=0.009), resulting in a greater delay between chemotherapy cycles. On the other hand, non-haematologic toxicities (nausea-vomiting, stomatitis and alopecia) were significantly lower with Epi-Vnr regimen than with FEC100. There were two deaths in the FEC100 arm during chemotherapy related to a poorly monitored septic shock and to a hypokaliemia resulting in major cardiac dysrhythmia. On the basis of our experience of FEC100 regimen, these fatal events remain rare in the regular use of this chemotherapy. Cardiac toxicities were not different between treatment arms. The number of cardiac events was identical and the incidence of decrease in LVEF during the follow-up period was 0.8 and 2.1%, respectively, without any development of subsequent cardiac complications or CHF. These results confirmed observations reported from our whole FASG database, which showed that the use of epirubicin-based adjuvant chemotherapy was associated with a low risk of left ventricular dysfunctions ([Fumoleau *et al*, 2006). When epirubicin was delivered within recommended doses, a favourable benefit/risk ratio was maintained.

Vinorelbine has a common target with taxanes, interfering with tubuline. However, the main difference in this mechanism is that vinorelbine inhibits the connection of microtubules, whereas taxanes promote the formation of microtubules and stabilise them. We have showed previously that a concomitant taxane–anthracycline regimen (TAC: docetaxel, doxorubicin and cyclophosphamide) did not provide different outcomes from FEC100 in a side-by-side comparison involving node-positive (more than three positive auxillary lymph nodes) breast cancer patients (Fumoleau *et al*, 2003). Results of the present study confirmed the similar efficacy between FEC100 and a regimen including an anthracyline delivered concomitantly with a drug interfering with tubuline. On the other hand, a sequential treatment of FEC100 (three cycles) followed by docetaxel (three cycles) provided a significant improvement in DFS and OS compared with six cycles of FEC100 for node-positive breast cancer patients (Roché *et al*, 2004). Thus, the best way to use vinorelbine in adjuvant setting could be a sequential regimen with an anthracycline, as it has been shown with docetaxel and paclitaxel (Henderson *et al*, 2003; Roché *et al*, 2004; Mamounas *et al*, 2005; Crown *et al*, 2006). A recent trial compared docetaxel and vinorelbine followed by FEC with or without trastuzumab in node-positive or high-risk node-negative early breast cancer patients (Joensuu *et al*, 2006). After 3 years of follow-up, docetaxel significantly improved recurrence-free survival compared to vinorelbine without improvement in survival. Docetaxel was associated with more adverse events than vinorelbine, and noteworthy, trastuzumab therapy did not lead to an increase risk of LVEF decrease or CHF.

The Epi-Vnr regimen provided a similar efficacy to the classical FEC100 regimen and could be an alternative option for node-positive breast cancer patients. The choice of adjuvant chemotherapy should be based on safety and patient's preferences. Knowing the interest of sequential adjuvant chemotherapy, further trials evaluating a regimen in which FEC100 was used sequentially with vinorelbine as well as a direct comparison with a taxane-based regimen could be of interest.

## Figures and Tables

**Table 1:** Patient and tumour characteristics at baseline

**Characteristics *n* (%)**	**FEC100 (*n*=241)**	**Epi-Vnr (*n*=241)**	***P*-value**
*Age (years)*			
Median (range)	50 (24–69)	51 (29–66)	0.61
<40	35 (14.5)	29 (12.0)	0.42
⩾40	206 (85.5)	212 (88.0)	
			
*Menopausal status*			
Premenopausal	115 (47.7)	117 (48.5)	0.85
Postmenopausal	126 (52.3)	124 (51.5)	
			
*Surgery*			
Tumorectomy	144 (59.8)	156 (64.7)	0.34
Mastectomy	96 (39.8)	85 (35.3)	
Unknown	1 (0.4)	0	
			
*Histology*			
Ductal	189 (78.4)	199 (82.6)	0.08
Lobular	32 (13.3)	17 (7.1)	
Other	13 (5.4)	12 (5.0)	
Unknown	7 (2.9)	13 (5.4)	
			
*SBR grade*			
1	17 (7.1)	14 (5.8)	0.33
2	92 (38.2)	90 (37.3)	
3	117 (48.5)	111 (46.1)	
Unknown/not gradable	15 (6.2)	26 (10.8)	
			
*Histological tumour size (mm)*			
⩽20	83 (34.4)	93 (38.6)	0.64
>20	132 (54.8)	123 (51.0)	
Unknown	26 (10.8)	25 (10.4)	
			
*Axillary lymph node involvement*			
1–3	56 (23.2)	63 (26.1)	0.46
>3	185 (76.8)	178 (73.9)	
			
*Hormone-receptor status*			
Positive (ER and/or PgR)	79 (32.8)	97 (40.2)	0.15
Negative (ER and PR)	152 (63.1)	131 (54.4)	
Unknown	10 (4.1)	13 (5.4)	

Epi-Vnr=epirubicin–vinorelbine; ER=estrogen receptors; PgR=progesterone receptors; SBR=Scarff, Bloom and Richardson.

**Table 2:** Treatment characteristics

**Characteristics**	**FEC100**	**Epi-Vnr**
Treated patients (*n*)	236	233
Cycles delivered (*n*)	1386	1373
Six cycles completed, no. of treated patients (%)	224 (94.9)	224 (96.1)
Treatement delayed, no. of cycles (%)	355 (25.6)	422 (30.7)
		
*Cumulative dose, median (range)*		
Epirubicin	600 (100–628)	300 (97–379)
Vinorelbine	NA	300 (51–387)
		
*Mean relative dose intensity, % (s.d.)*		
Epirubicin	89.1 (11.8)	88.9 (10.2)
Vinorelbine	NA	87.8 (11.2)
		
*Mean dose intensity, mg m*^*−2*^* week*^*−1*^ *(s.d.)*		
Epirubicin	29.9 (3.9)	15.1 (1.5)
Vinorelbine	NA	14.9 (1.7)

Epi-Vnr=epirubicin–vinorelbine; NA=not applicable; s.d.=standard deviation.

**Table 3:** Summary of events in patients entered in the efficacy analysis

**Events *n* (%)**	**FEC100 (*n*=235)**	**Epi-Vnr (*n*=236)**
*First event*	105 (44.7)	110 (46.6)
Relapse	87 (37.0)	95 (40.2)
Local only	13 (5.5)	17 (7.2)
Distant (with or without local)	74 (31.5)	78 (33.0)
Contralateral breast cancer	10 (4.2)	5 (2.1)
Second cancer	5 (2.1)	5 (2.1)
Death	3 (1.3)	5 (2.1)
		
*Any event*		
Relapse	89 (37.9)	96 (40.7)
Local only	10 (4.2)	9 (3.8)
Local then distant	4 (1.7)	8 (3.4)
Local and distant simultaneously	5 (2.1)	8 (3.4)
Distant only	70 (29.8)	71 (30.1)
Contralateral breast cancer	10 (4.2)	10 (4.2)
Second cancer	8 (3.4)	5 (2.1)
Any death	62 (26.4)	71 (30.1)
Of breast cancer	54 (23.0)	63 (26.7)
Of second cancer	3 (1.3)	0
Due to toxic effects	1 (0.4)	0
Due to cardiac events	2 (0.8)	1 (0.4)
Other causes	2 (0.8)^a^	7 (3.0)^b^

Epi-Vnr=epirubicin–vinorelbine.

a Necrotic enteritis (*n***=**1), unknown (*n***=**1).

b Cirrhosis (*n*=1), unknown (*n*=6).

**Table 4:** Prognostic factors of relapse

	**Univariate analysis**		**Multivariate analysis**	
**Prognostic factors**	**HR (95% CI)**	***P*-value**	**HR (95% CI)**	***P*-value**
*Chemotherapy*				
FEC100	0.90 (0.61–1.19)	0.47	0.91 (0.58–1.24)	0.57
Epi-Vnr	1		1	
				
*Tamoxifen*				
No	1.52 (1.21–1.83)	0.009	1.34 (0.82–1.86)	0.26
Yes	1		1	
				
*Age (years)*				
<40	1.58 (1.20–1.96)	0.01	1.41 (0.91–1.91)	0.17
⩾40	1		1	
				
*Menopausal status*				
Premenopausal	1.48 (1.19–1.77)	0.008	1.15 (0.63–1.67)	0.59
Postmenopausal	1		1	
				
*Surgery*				
Tumorectomy	0.76 (0.47–1.05)	0.06	0.68 (0.32–1.04)	0.04
Mastectomy	1		1	
				
*SBR grade*		0.07		0.21
1	0.55 (0.00–1.23)		0.56 (0.00–1.31)	
2	0.75 (0.43–1.07)		0.79 (0.42–1.16)	
3	1		1	
				
*Histological tumour size (mm)*				
⩽20	0.66 (0.34–0.98)	0.01	0.68 (0.31–1.05)	0.04
>20	1		1	
				
*Lymph node involvement*				
1–3	0.65 (0.29–0.99)	0.02	0.84 (0.29–1.39)	0.52
>3	1		1	
				
*HR status*				
Negative	0.89 (0.58–1.20)	0.45	0.92 (0.47–1.37)	0.73
Positive	1		1	

95% CI=95% confidence interval; Epi-Vnr=epirubicin–vinorelbine; HR=hazard ratio; HR=hormone receptors; SBR=Scarff, Bloom and Richardson.

**Table 5:** Haematologic and non-haematologic adverse events per treated patient

**Adverse events *n* (%)**	**FEC100 (*n*=236)**	**Epi-Vnr (*n*=233)**	***P*-value**
*Neutropenia on day 21*			
Grades 1–2	105 (44.5)	118 (50.6)	0.009
Grades 3–4	41 (17.4)	56 (24.0)	
			
*Infection*			
Grades 1–2	71 (30.1)	67 (28.8)	0.95
Grades 3–4	5 (2.1)^a^	5 (2.1)	
			
*Anemia*			
Grades 1–2	95 (40.2)	93 (39.9)	0.60
Grade 3	3 (1.3)	1 (0.4)	
			
*Nausea-vomiting*			
Grades 1–2	150 (63.6)	151 (64.8)	<0.0001
Grades 3–4	58 (24.6)	27 (11.6)	
			
*Stomatitis*			
Grades 1–2	85 (36.0)	49 (21.0)	0.0007
Grades 3	7 (3.0)	4 (1.7)	
			
*Diarrhoea*			
Grade 1–2	30 (12.7)	24 (10.3)	0.27
Grade 3	0 (0.0)	2 (0.9)	
			
*Alopecia*			
Grades 1–2	49 (20.8)	113 (48.5)	<0.0001
Grade 3	160 (67.8)	64 (27.5)	
			
*Asthenia*			
Grades 1–2	47 (19.9)	38 (16.3)	0.14
Grade 3	5 (2.1)	1 (0.4)	

Epi-Vnr=epirubicin–vinorelbine.

a One patient died from a septic shock.
